# Supplementary material for: Using DNA metabarcoding and a novel canid-specific blocking oligonucleotide to investigate the composition of animal diets of raccoon dogs (Nyctereutes procyonoides) inhabiting the waterside area in Korea
Source: PLoS One. 2022 Jul 25;17(7):e0271118. doi: 10.1371/journal.pone.0271118 (PMC9312373; doi:10.1371/journal.pone.0271118)
Supplement: S1 File — (PDF) [file pone.0271118.s001.pdf]

Supporting Information for:

Using DNA metabarcoding and a novel canid-specific blocking oligonucleotide to investigate the composition of animal diets of raccoon dogs (*Nyctereutes procyonoides*) inhabiting the waterside area in Korea

Short title: DNA metabarcoding for raccoon dogs

Cheolwoon Woo[1], Priyanka Kumari[1,2], Kyung Yeon Eo [3*], Woo-Shin Lee [4], Junpei Kimura [5] and Naomichi Yamamoto[1,2*]

1. Department of Environmental Health Sciences, Graduate School of Public Health, Seoul National University, Seoul 08826, Republic of Korea
2. Institute of Health and Environment, Graduate School of Public Health, Seoul National University, Seoul 08826, Republic of Korea
3. Department of Animal Health and Welfare, College of Healthcare and Biotechnology, Semyung University, Jecheon 27136, Republic of Korea
4. Department of Forest Sciences, College of Agriculture and Life Science, Seoul National University, Seoul 08826, Republic of Korea
5. College of Veterinary Medicine, Seoul National University, Seoul 08826, Republic of Korea

*Correspondence

E-mail: vetinseoul@semyung.ac.kr (KYE)

E-mail: nyamamoto@snu.ac.kr (NY)

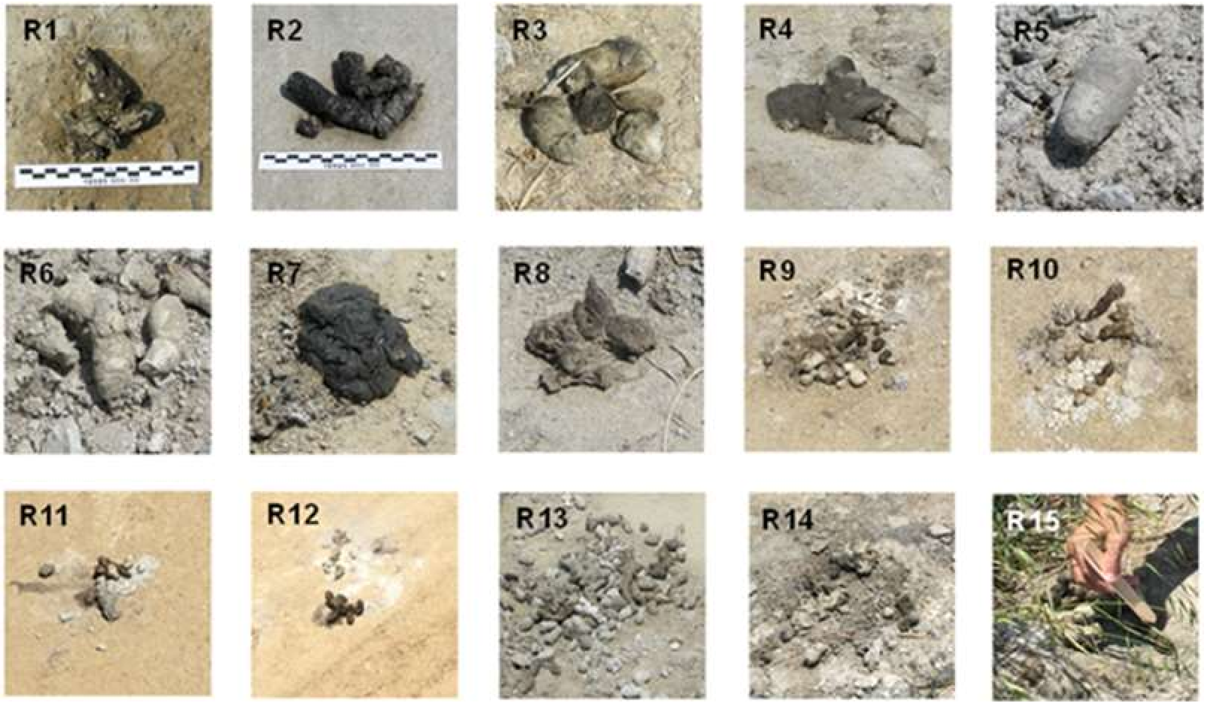

S1 Fig. Fecal samples of raccoon dogs. The samples were collected from the Seosan reclaimed paddy fields in Seosan city in Chungcheongnam-do, South Korea.

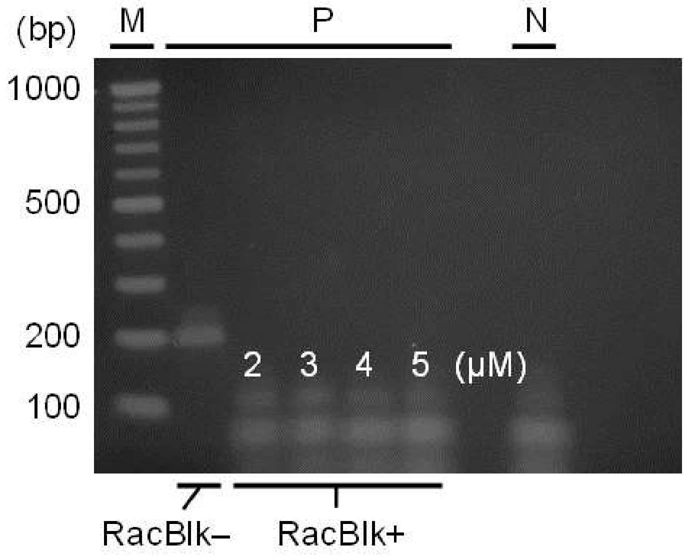

S2 Fig. Vertebrate-specific PCR assay with and without the blocking oligonucleotide RacBlk. Standard DNA extracted from a raccoon dog tissue sample was used as a template, and four concentrations of RacBlk of 2, 3, 4, and 5 μM were tested. Abbreviations: M, DNA marker; RacBlk–, without RacBlk, RacBlk+, with RacBlk; P, positive control; and N, negative control.

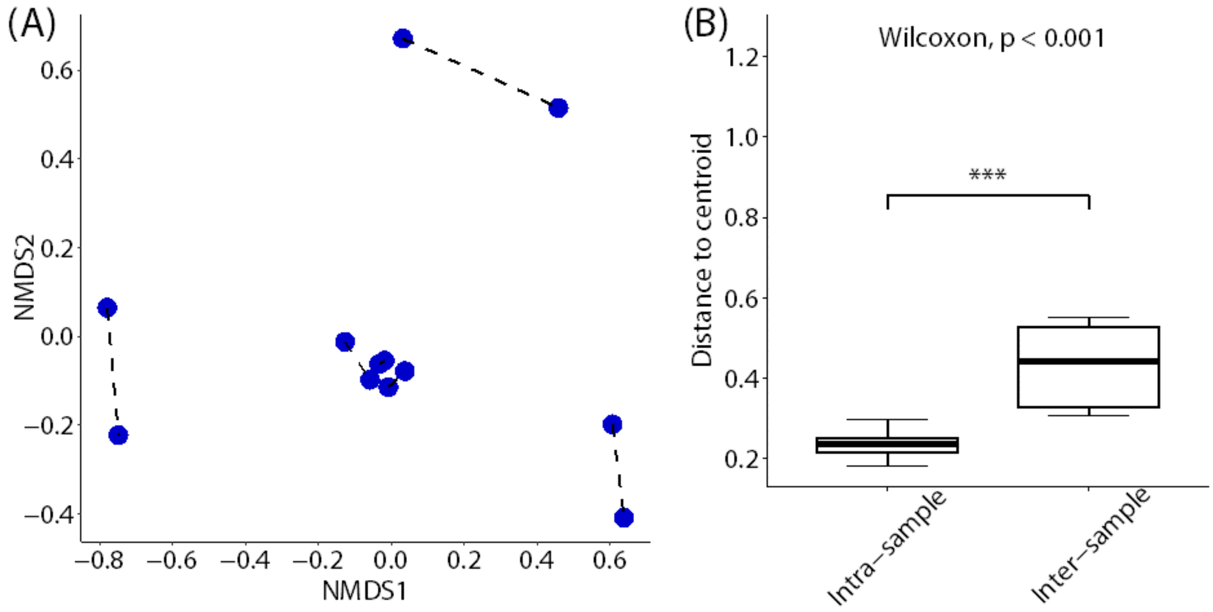

S3 Fig. Reproducibility of dietary composition characterized by technical duplicates. The data obtained by the vertebrate 12S rRNA sequencing with RacBlk are shown. (A) Non-metric multidimensional scaling (NMDS) plot showing the Bray–Curtis dissimilarity of composition of prey animals. The data from the same sample are connected by a line. (B) Boxplot showing the intra- and inter-sample variances of prey composition. The three asterisks (***) represent $p < 0.001$ by the Wilcoxon rank-sum test.

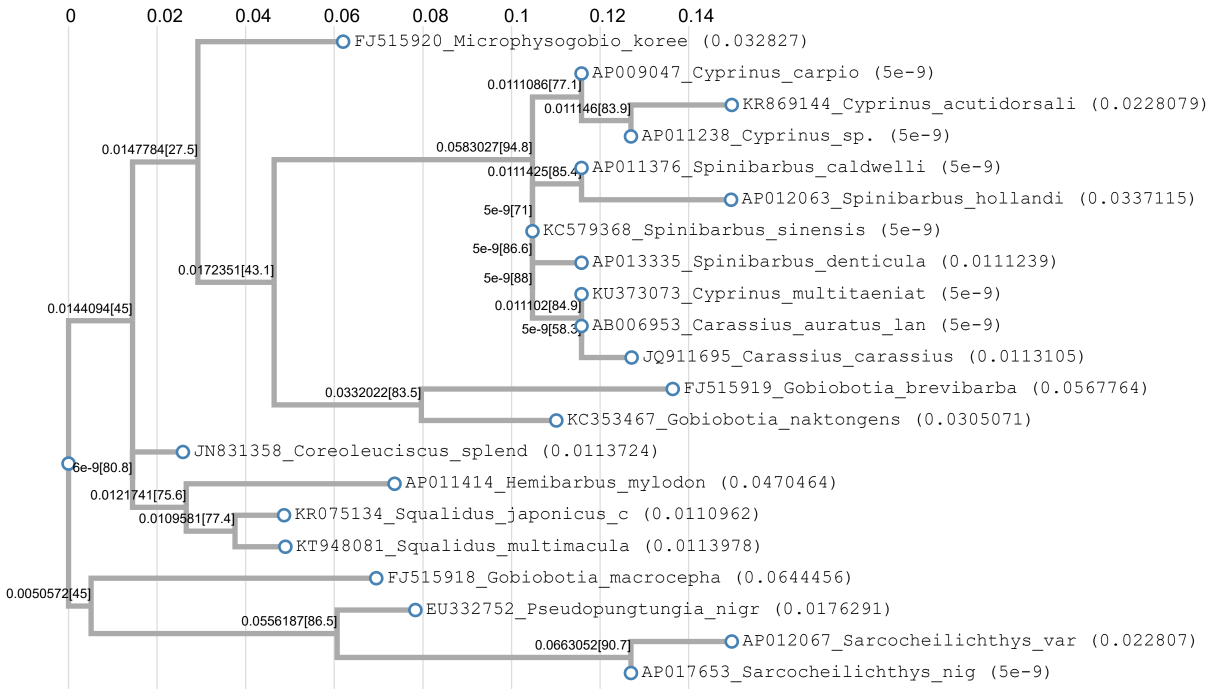

S4 Fig. Comparison of sequence similarity among species of the family Cyprinidae. All of sequences of Cyprinidae species belonging to the genera *Cyprinus*, *Carassius* and *Spinibarbus* and the genera that are known to be endemic in Korea were retrieved and aligned. For the alignment, the sequences were trimmed to the targeted amplified region of primers 12SV5F and 12SV5R. Multiple sequence alignment was performed by CLUSTALW (https://www.genome.jp/tools-bin/clustalw) with slow and accurate pairwise alignment method and default parameter settings. The FastTree method was used.

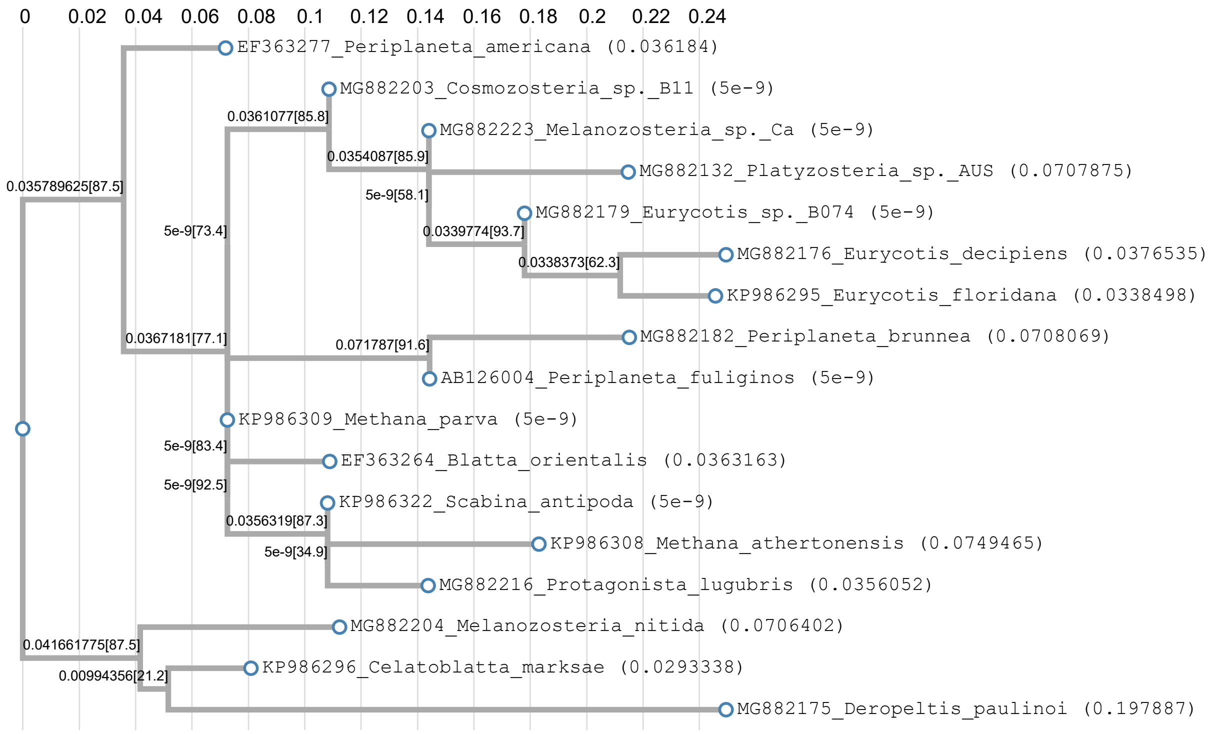

S5 Fig. Comparison of sequence similarity among species of the family Blattidae. All of sequences of species belonging to the familly Blattidae were retrieved from the reference database and aligned. For the alignment, the sequences were trimmed to the targeted amplified region of primers 16SMAV-F and 16SMAV-R. Multiple sequence alignment was performed by CLUSTALW (https://www.genome.jp/tools-bin/clustalw) with slow and accurate pairwise alignment method and default parameter settings. The FastTree method was used.

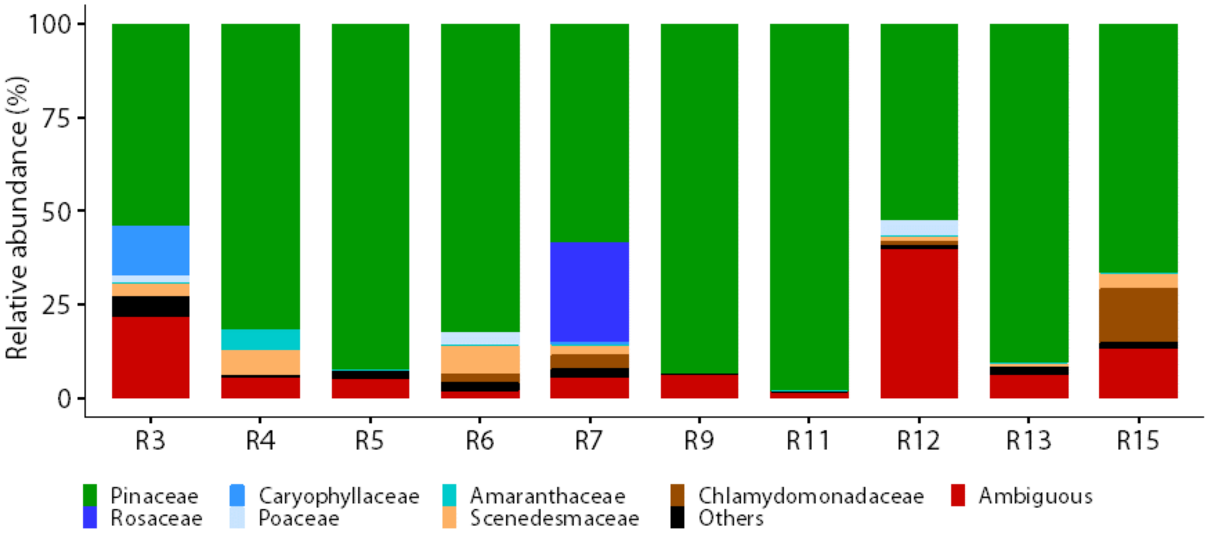

S6 Fig. Relative abundance of plants identified at the family level. The data were obtained by sequencing the internal transcribed spacer 2 (ITS2) region with universal plant-specific primers ITS-p3 and ITS-u4 [1]. The analyses were performed mainly with USEARCH v.11.0.667 [2] according to the previously reported method [3]. Briefly, taxonomic assignment was performed by SINTAX algorithm of USEARCH with 0.5 of a cutoff value [4] against the ITS2 database [5, 6]. Sequencing statistics and accession number of raw sequences are listed in S3 Table.

S1 Table. Metadata of fecal samples.

Sample ID	Sampling date	Sampling location		Origin [a]
	YYYY/MM/DD	Latitude	Longitude	
R3	2017/05/21	36°40'34.80"N	126°28'10.85"E	Raccoon dog
R4	2017/05/21	36°40'07.82"N	126°28'40.65"E	Raccoon dog
R5	2017/05/21	36°39'59.61"N	126°28'46.67"E	Raccoon dog
R6	2017/05/21	36°40'18.71"N	126°29'13.25"E	Raccoon dog
R7	2017/05/21	36°40'15.01"N	126°30'25.57"E	Raccoon dog
R9	2017/05/21	36°39'39.54"N	126°27'49.62"E	Raccoon dog
R10	2017/05/21	36°39'41.34"N	126°27'55.72"E	Raccoon dog
R11	2017/05/21	36°39'45.32"N	126°28'08.19"E	Raccoon dog
R12	2017/05/21	36°39'47.84"N	126°28'16.84"E	Raccoon dog
R13	2017/05/21	36°37'50.97"N	126°29'28.88"E	Raccoon dog
R15	2017/05/21	36°37'44.85"N	126°28'37.22"E	Raccoon dog
R1	2017/05/21	36°42'40.86"N	126°28'14.45"E	n.a.
R2	2017/05/21	36°40'48.54"N	126°27'48.91"E	n.a.
R8	2017/05/21	36°40'56.67"N	126°29'49.92"E	n.a.
R14	2017/05/21	36°37'48.87"N	126°28'48.62"E	n.a.

[a] The origin of fecal samples were identified by the raccoon dog specific PCR assay.
Abbreviation: n.a., not amplified by the raccoon dog-specific PCR assay.

S2 Table. Sequencing statistics. The statistics of vertebrate 12S rRNA gene sequencing and invertebrate 16S rRNA gene sequencing is shown.

Sample ID	12S rRNA gene of vertebrates without RacBlk		12S rRNA gene of vertebrates with RacBlk		16S rRNA gene of invertebrates	
	Accession number	No. sequence reads	Accession number	No. sequence reads	Accession number	No. sequence reads
R3	SAMN26117575	119,583	SAMN26117564	161,761	SAMN26117586	76,834
R3_dup	-	-	SAMN26117606	169,293	-	-
R4	SAMN26117576	121,867	SAMN26117565	164,162	SAMN26117587	81,379
R4_dup	-	-	SAMN26117607	178,742	-	-
R5	SAMN26117577	132,174	SAMN26117566	158,509	SAMN26117588	80,272
R5_dup	-	-	SAMN26117608	146,458	-	-
R6	SAMN26117578	90,684	SAMN26117567	159,838	SAMN26117589	84,111
R7	SAMN26117579	118,744	SAMN26117568	128,580	SAMN26117590	73,268
R7_dup	-	-	SAMN26117609	133,462	-	-
R9	SAMN26117580	140,180	SAMN26117569	179,233	SAMN26117591	92,981
R10	SAMN26117581	97,605	SAMN26117570	134,897	n.a.	n.a.
R11	SAMN26117582	141,697	SAMN26117571	147,294	SAMN26117592	81,507
R12	SAMN26117583	124,751	SAMN26117572	127,689	SAMN26117593	138,308
R12_dup	-	-	SAMN26117610	116,491	-	-
R13	SAMN26117584	144,171	SAMN26117573	144,589	SAMN26117594	5,338
R15	SAMN26117585	135,615	SAMN26117574	143,413	SAMN26117595	81,629
R15_dup	-	-	SAMN26117611	118,944	-	-
Total		1,367,071		2,513,355		795,627

Abbreviation: RacBlk, blocking oligonucleotide for the raccoon dog; n.a., not amplified by PCR.
Symbol: -, duplicate unavailable.

S3 Table. Sequencing statistics of plant internal transcribed spacer (ITS) sequencing.

Sample ID	Accession number	No. sequence reads
R3	SAMN26117596	40,673
R4	SAMN26117597	34,750
R5	SAMN26117598	21,023
R6	SAMN26117599	12,230
R7	SAMN26117600	24,467
R9	SAMN26117601	23,048
R10	n.a.	n.a.
R11	SAMN26117602	19,482
R12	SAMN26117603	19,099
R13	SAMN26117604	19,097
R15	SAMN26117605	20,678
Total		234,547

Abbreviation: n.a., not amplified by PCR.

Supporting References

1. Cheng T, Xu C, Lei L, Li C, Zhang Y, Zhou S. Barcoding the kingdom Plantae: new PCR primers for ITS regions of plants with improved universality and specificity. Mol Ecol Resour. 2016;16(1):138-49.
2. Edgar RC. Search and clustering orders of magnitude faster than BLAST. Bioinformatics. 2010;26(19):2460–1.
3. Dong K, Woo C, Yamamoto N. Plant assemblages in atmospheric deposition. Atmos Chem Phys. 2019;19(18):11969–83.
4. Edgar RC. Accuracy of taxonomy prediction for 16S rRNA and fungal ITS sequences. PeerJ. 2018;6:e4652.
5. Sickel W, Ankenbrand MJ, Grimmer G, Holzschuh A, Härtel S, Lanzen J, et al. Increased efficiency in identifying mixed pollen samples by meta-barcoding with a dual-indexing approach. BMC Ecol. 2015;15(1):20.
6. Ankenbrand MJ, Keller A, Wolf M, Schultz J, Förster F. ITS2 Database V: Twice as Much. Molecular Biology and Evolution. 2015;32(11):3030-2.
